# Perspective: an optoelectronic future for heterogeneous, dendritic computing

**DOI:** 10.3389/fnins.2024.1394271

**Published:** 2024-04-17

**Authors:** Luis El Srouji, Mahmoud Abdelghany, Hari Rakul Ambethkar, Yun-Jhu Lee, Mehmet Berkay On, S. J. Ben Yoo

**Affiliations:** Department of Electrical and Computer Engineering, University of California, Davis, Davis, CA, United States

**Keywords:** neuromorphic computing, silicon photonic computing, dendritic computing, heterogeneous computing, analog computing

## Abstract

With the increasing number of applications reliant on large neural network models, the pursuit of more suitable computing architectures is becoming increasingly relevant. Progress toward co-integrated silicon photonic and CMOS circuits provides new opportunities for computing architectures with high bandwidth optical networks and high-speed computing. In this paper, we discuss trends in neuromorphic computing architecture and outline an optoelectronic future for heterogeneous, dendritic neuromorphic computing.

## 1 Introduction

Carver Mead introduced the term “neuromorphic” in 1990 in an invited article where he explained the inherent wastefulness of digital computation. In brief, he argued that each operation in a digital system requires the switching of about 10,000 transistors and that much of the power required to switch these transistors is actually due to the excess capacitance on each gate caused by wiring between each transistor. To reduce these problems, Mead (1990) argued that computing algorithms should be designed for less data movement and that engineers should use the natural properties of devices to perform various operations. Despite these arguments, the success of digital computers based on the von Neumann architecture continued to grow and dominate the market into the present day (Backus, 1978).

A human brain, on the other hand, is a highly parallelized computing system whose analog dynamics offer many advantages for high-performance computing. It is estimated that the human brain can process up to 10^23^ operations every second compared to the roughly 10^9^ operations per second possible with a traditional computer based on the von Neumann architecture (Thagard, 2002). Despite this fact, a desire for deterministic components has enforced a preference for digital circuits in computer architecture. Meanwhile, biological neural networks are surprisingly noise-tolerant despite synaptic efficacies as low as 20% (Stevens and Wang, 1994). Nonetheless, as the trend of Moore's Law (Theis and Wong, 2017) wanes, further advancements in computing can no longer rely on increasing transistor speeds and density. As a result, general-purpose computing systems are expected to be increasingly replaced by application-specific integrated circuits (ASICs) in various compute-intensive applications, including neural networks (Solli and Jalali, 2015; Ranganathan, 2020). While vectorized tensor processing units (TPUs) and graphical processing units (GPUs) have made traditional deep neural network (DNN) architectures more practical on digital systems, carefully designed analog and mixed-signal ASICs can often offer improvements to throughput and system latency while reducing power consumption.

Recent progress in the area of silicon photonics has pushed industry leaders such as GlobalFoundries to develop a co-integrated process design kit (PDK)—labeled GF 45SPCLO—that allows circuit designers to place photonic elements and CMOS circuits on the same physical substrate (Rakowski et al., 2020). This process opens new doors for optoelectronic ASICs that employ silicon photonic elements for high-bandwidth data communication networks (Beausoleil, 2011) alongside CMOS electronic circuits for high-speed computing structures (Hassan et al., 2023).

In the following manuscript, we will highlight two important features of biological neural networks from the perspective that co-integrated photonic and electronic technologies are key to the future of neuromorphic computing. Section 2 will begin by describing the advantages of heterogeneous neural dynamics and discuss the limited number of neuromorphic devices that incorporate this heterogeneity. Next, Section 3 will discuss the computational properties of biological dendrites and review existing approaches to implement dendritic computing. Finally, we discuss a proposed optoelectronic chiplet architecture that is capable of supporting these features in a scalable neuromorphic system.

## 2 Heterogeneous neural dynamics

**Figure 1:**
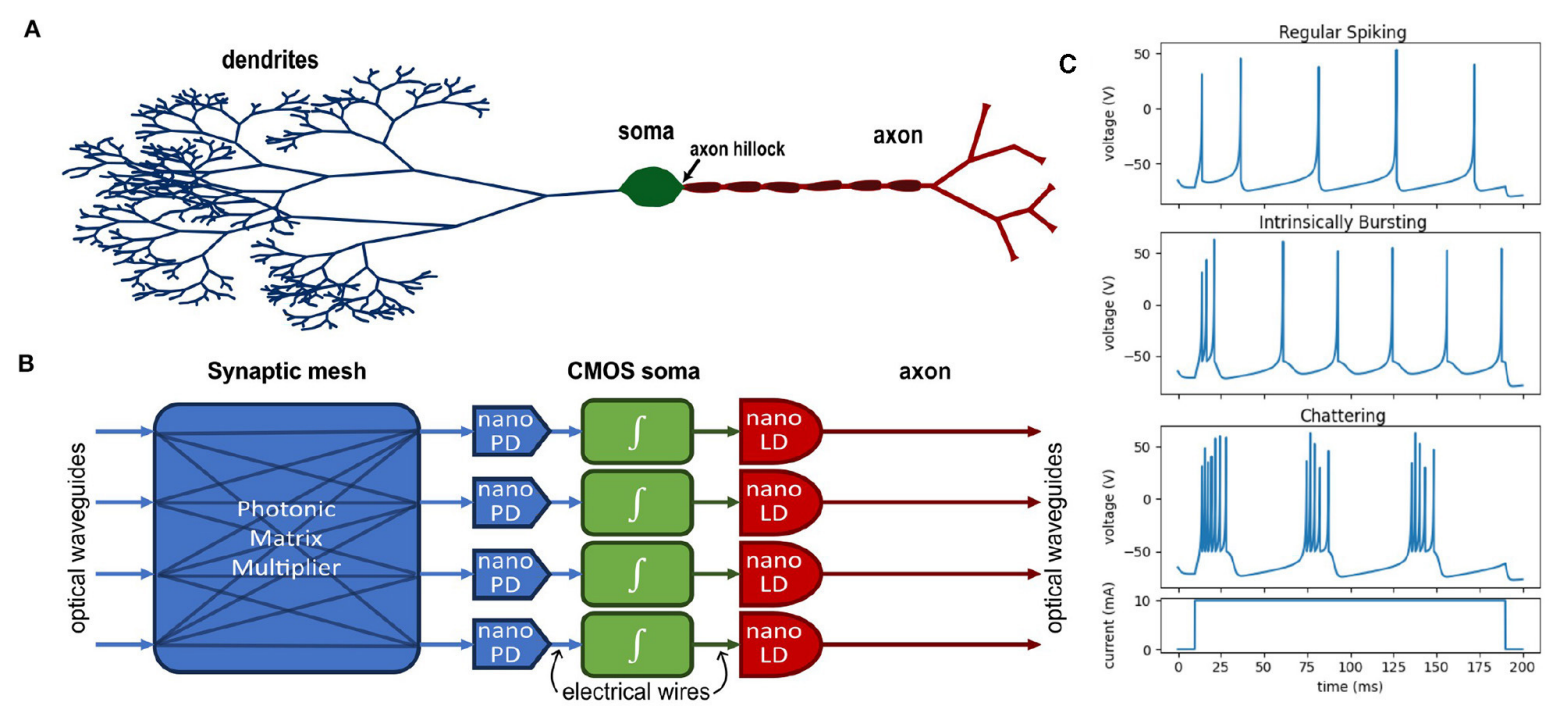
**(A)** Diagram of a biological neuron compared to **(B)** a four-neuron layer of an optoelectronic neural network—a nanophotodetector (nano PD) and nanolaser (nano LD) perform signal transduction. **(C)** Examples of various neural dynamics according to the Izhikevich neuron model (Izhikevich, 2003).

Artificial neural networks employ a variety of nonlinear transformations (activation functions) to guide a model into choosing an efficient encoding for a given task (Rasamoelina et al., 2020; Mercioni and Holban, 2023). While artificial neural networks are considered to be universal function approximators (Hornik et al., 1989; Lu et al., 2017), it is well known empirically that the choice of nonlinearity can be pivotal to the success of the DNN. Similarly, it is known that biological neurons display a wide range of dynamic behaviors—see Figure 1C for examples of three typical behaviors of cortical neurons according to the Izhikevich model (Izhikevich, 2003). However, when designing hardware accelerators for neuromorphic computing, engineers must decide what level of specificity or generality is needed to support the various neural dynamics required for the most common DNNs. Following Mead's argument (Mead, 1990), it is far more efficient to use the natural physical properties of a device to provide nonlinear behavior, but these properties are often fixed after fabrication and impossible to program. Even in the case of digital systems, general-purpose computing elements necessarily consume more physical resources and power in order to serve the generic case.

For example, Intel's Loihi processor (Davies et al., 2018) is designed as a many-core digital system where each “neurocore” asynchronously updates a set of internal variables using a limited digital core. In the first iteration of the chip, the internal updates followed a fixed schedule and computed discrete updates to a current-based, leaky-integrate-and-fire (CUBA LIF) model that was not programmable. Loihi 2, however, included a micro-code programmable neuron that allows an arbitrary neuron model to be implemented as long as its instructions fit in the core's local memory (Orchard et al., 2021). Each core on Loihi 2 has the same memory size and must contain all the necessary parameters for neurons and synapses on that core. This means that more complicated neuron models limit the maximum neuron density of the core, and neurons which use different micro-code models must be implemented on separate neurocores. Nonetheless, Intel invested in this generality despite the additional implementation complexity because of the expectation that heterogeneous neural dynamics would hold several key computational benefits.

Biological neural networks are remarkably heterogeneous regarding individual neuron dynamics and morphological structure. Koch and Laurent (1999) argues that this heterogeneity is a direct consequence of the complexity of behaviors and sensory modalities that the brain must handle. To establish whether this heterogeneity is advantageous or purely an evolutionary epiphenomenon, several analyses have compared neural network structures with and without heterogeneity and shown that the variability of neural responses in heterogeneous populations increases the sensitivity of a population code and, therefore, improves the precision at which it can be read out (Shamir and Sompolinsky, 2006; Chelaru and Dragoi, 2008; Marsat and Maler, 2010). Population codes are often associated with sensory stimuli because of their ability to handle noisy input (Averbeck et al., 2006), and these results show that heterogeneity may be key to balancing sensitivity and signal-to-noise ratio. Perez-Nieves et al. (2021) also showed that the heterogeneity of synaptic time constants in a reservoir network improved generalization, robustness to hyperparameters, and overall learning performance. While these results were demonstrated by digital simulation, Mead's argument should remind us that an architecture that uses the natural dynamics of its computing elements would be more efficient than a digital emulator to implement a heterogeneous neural network.

### 2.1 Optoelectronic neurons

A number of efforts have been made to design analog photonic neurons by drawing a bijection between semiconductor laser and amplifier dynamics and the dynamics of a LIF neuron model (Tait et al., 2014; Prucnal et al., 2016). These efforts are motivated by the advantages in bandwidth and throughput of silicon photonic interconnects (Miller, 2000, 2009; Agarwal et al., 2019; Huang et al., 2022)—a crucial advantage considering that human synaptic fan-out is on the order of 10,000 synapses per neuron. However, optical dynamics are controlled by material parameters that are fixed after device fabrication and are mostly unprogrammable (i.e., carrier lifetimes in the gain medium of a laser). As a result, photonic neurons may not be sufficient to replicate the breadth of heterogeneity found in biological neural networks. An optoelectronic approach, however, may be more feasible.

Electronics are preferable for designing programmable circuits given the long history of well-developed design principles for CMOS circuits, and many such programmable circuit models have been demonstrated (Indiveri et al., 2011). An optoelectronic neuron would combine this programmability with the benefits of optical interconnects. Under this approach, photodetectors transduce optical signals into electrical currents and are analogous to the synaptic receptors in a biological neuron. These currents are collected by a capacitor in the circuit analogous to the membrane capacitance. A CMOS circuit behaves like the neuron soma and provides the feedback dynamics that generate the neuron's excitable (spiking) behavior. In the biological neuron, the membrane potential is only propagated to the axon when the activity near the axon hillock reaches a threshold; similarly, in the optoelectronic neuron, a CMOS amplifier drives a laser only when the neuron is spiking. Figures 1A, B show a functional comparison between a biological neuron (A) and a block diagram of the optoelectronic neuron (B).

Few have demonstrated such optoelectronic neurons with spiking dynamics, and those existing implementations show limited or no programmable dynamics (Balle et al., 2013; Tait et al., 2015). More recently, Lee et al. (2024) demonstrated a programmable spiking optoelectronic neuron using the GF 45SPCLO PDK. However, because of the lack of on-chip lasers available in this process, an off-chip vertical cavity laser was externally connected to the neuron. The neuron efficiency was projected to improve on a more advanced CMOS node and with an on-chip micro-scale laser such as a low-threshold ring laser (Liang et al., 2016). Despite the strengths of this optoelectronic approach, a new process that can reliably integrate on-chip lasers alongside these CMOS and silicon photonic circuits is required to make packaging more feasible. Existing implementations of photonic matrix multipliers have large footprints, where each matrix element requires a roughly 900 μ*m*^2^ area (Ramey, 2020; Feldmann et al., 2021). This limits the number of synaptic connections and neurons that are available on a single chip. As such, an advanced packaging scheme is needed for photonic and optoelectronic neural networks to be practical at the scales of modern DNNs. A 3D photonic-electronic packaging scheme has been proposed for this purpose in which chiplets are stacked and tiled onto an interposer using a combination of 3D photonic and electronic interconnects (Zhang et al., 2020). Early results have been demonstrated in other application contexts (Chang et al., 2023); however, a complete photonic-electronic neuromorphic chiplet network has not yet been demonstrated.

## 3 Dendritic computing

Despite the variety of nonlinearities mentioned in Section 2, the vast majority of DNNs rely on a point-neuron model that lacks the temporal and spatial complexity of a biological neuron. Under this limited model, synaptic integration and nonlinearities across the network are considered instantaneous. Meanwhile, biological neurons vary so widely in morphology and dynamics that a standard taxonomy for neuron classification has yet to be established (Zeng and Sanes, 2017). For example, pyramidal neurons in Layer V are distinct from those in Layer II/III of the human cortex and carry distinct properties for synaptic integration even within the same cortical area (Spruston, 2008). This diversity in biological networks has led to the hypothesis that the increased spatio-temporal complexity could be related to several major advantages of biological neural networks over the modern implementation of DNN (Acharya et al., 2022).

**Figure 2:**
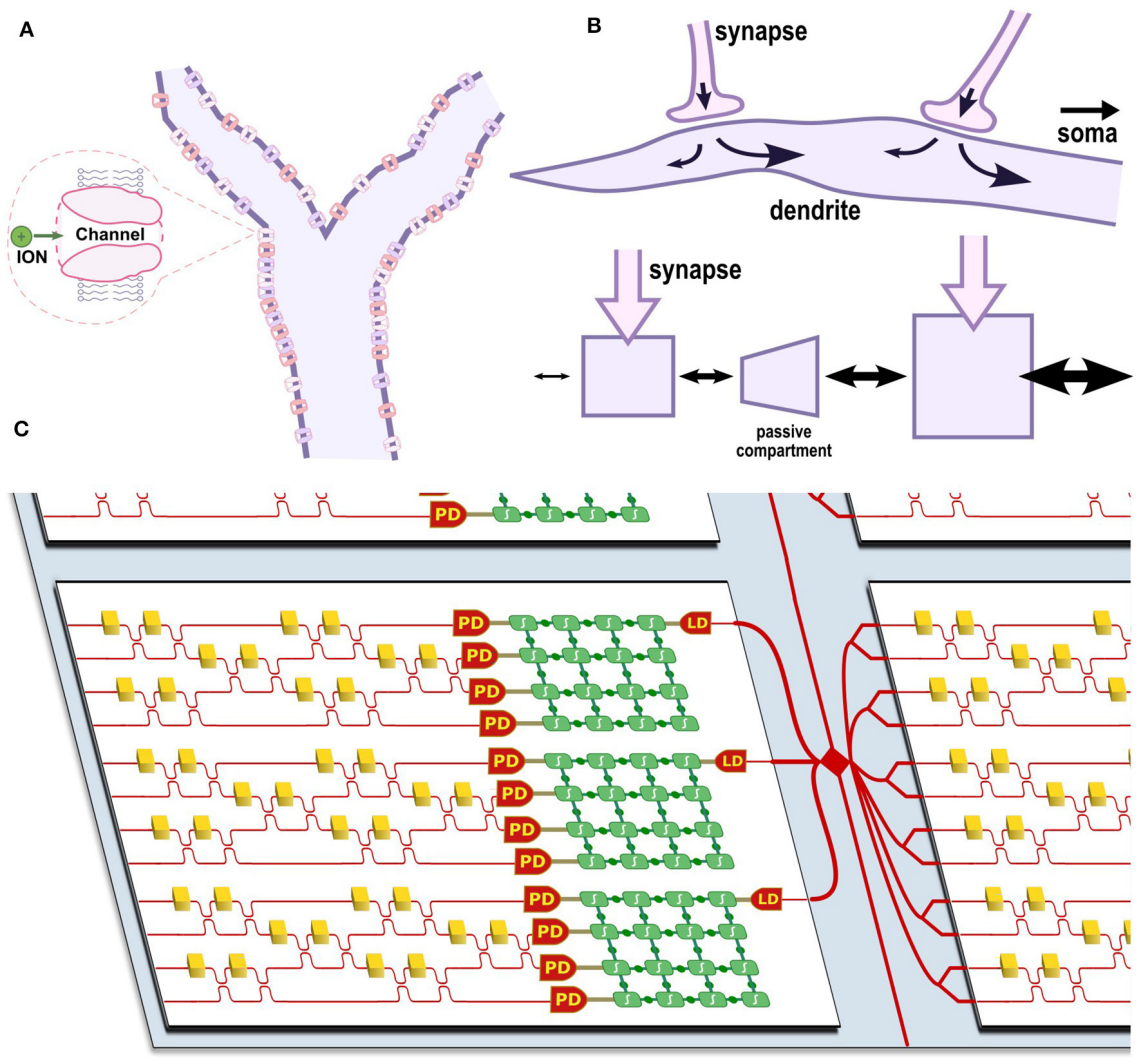
**(A)** Diagram of a dendritic branch with various ion channels. **(B)** Diagram of current flow between two synapses along a dendrite and an equivalent multi-compartment model where current flow is indicated by arrow size. **(C)** Diagram of a photonic-electronic chiplet architecture for optoelectronic dendritic computing. Each chiplet (white) shows several multi-compartment optoelectronic neurons and is coupled to other chiplets through an interposer (blue) containing an inter-chip routing network (red). The neurons contain multiple programmable CMOS blocks (green) interconnected via a switch matrix; leaf nodes receive optical input through a photodiode (PD), and root nodes emit optical output through a laser diode (LD). A Mach-Zehnder Interferometer mesh is shown as an example photonic synaptic mesh.

Various ion channels line the cell membrane along these dendritic branches (see Figure 2A), leading to both passive and active effects that can be modeled by cable theory (Koch, 1984). In the passive case, propagation along the dendrite is often compared to a lossy transmission line that propagates a signal with both attenuation and dispersion (Dayan and Abbott, 2001). Neurons with dendrite models are also more expressive than point neurons; Acharya et al. (2022) summarize three major features of dendrite models: weight plasticity, delay plasticity, and structural plasticity. Weight plasticity is the synaptic strength as modeled in DNNs. In contrast, the delay and structural plasticities are unique features of the dendrite that allow the dendrite to process sequences of information (discussed in Section 3.2).

### 3.1 Multi-compartment models

A multi-compartment model can be used to discretize the dendritic tree into localized segments under which the membrane dynamics are considered uniform. Such a model is necessary for neuromorphic computing because the cable dynamics of a biological neuron membrane are too complex to model in a continuous manner. Multiple software libraries such as the NEURON (Migliore et al., 2006) and Brian 2 (Stimberg et al., 2019) simulators have been written to model networks with such neurons. Each local compartment model includes active dynamics and membrane leakage currents, while a conductive channel models the diffusive, axial current flow between compartments. This model can be summarized by the following equation where *V*_*m*_ represents the localized membrane potential, *C*_*m*_ represents the localized membrane capacitance, *V*_*i*_ indexes the connected compartments, *g*_*k*_(*t*) indexes synaptic conductances, *E*_*k*_ indexes the reversal potential associated with a given synaptic conductance, and *F*(*V*_*m*_, *t*) summarizes the local membrane dynamics:


(1) $$\begin{array}{l} C_{m}\frac{dV_{m}}{dt}=F\left(V_{m},t\right)+\underset{k}{\sum }g_{k}\left(t\right)\left(E_{k}-V_{m}\right)+\underset{i}{\sum }g_{i}\left(V_{i}-V_{m}\right) \end{array}$$

Equation (1) highlights the similarities and differences between synaptic currents and dendritic currents. When a signal is received on the synapse, its conductance increases, and a current is generated according to the voltage difference between membrane potential and the equilibrium potential related to the given synaptic receptor. This equilibrium potential is often at the extrema of possible membrane potentials, and thus, a given synapse results in a polarizing or depolarizing current nearly exclusively.

In contrast, the conductance between dendritic compartments is time-independent, and the current flow direction can fluctuate depending on which compartment has the higher membrane potential at a given moment. The activity at each compartment is similar to the point model—a nonlinear response to a weighted sum—though the location of each compartment changes the efficacy and delay of its effect on the soma. By studying detailed models of hippocampal CA1 cells, Poirazi and Papoutsi (2020) showed that the complexity of these multi-compartment models could only be mapped onto a two-layer ANN, indicating that a single biological neuron is equivalent to a two-layer neural network. This result, however, only considers the rate-coded behavior of the neuron and does not fully capture the spike-timing sensitivity of dendritic models.

Biological dendrites are also tapered so that diffusive, axial currents preferentially flow in the feed-forward direction (toward the soma). This can be modeled as a decreasing resistance (increasing conductance) between compartments close to the soma. Figure 2B shows how this model captures the behavior of a dendrite with two synapses. The tapered end of the dendrite is shown on the left, and the increase in cross-sectional diameter toward the right of the dendrite allows more current flow toward the soma (not pictured) to the right. Note that the arrows shown in the figure indicate the directional preference for diffusive currents and do not represent static current flow. Similarly, the multi-compartment model has two compartments with synapses and a passive compartment in between that models the passive length of dendrite between the two synapses in the biological neuron. The following subsection discusses the consequences of this feature for sequence processing and highlights some early attempts at dendritic computing architectures.

### 3.2 Temporal complexity

Because the point neuron model lacks temporal dependence, machine learning tasks involving sequential data require carefully designed neural network models. Recurrent neural networks (RNNs) fake temporal complexity and memory by applying the network repeatedly for some number of simulated time steps (Elman, 1991; Lipton et al., 2015). Information is retained in memory based on feedback pathways, but each point neuron could instantaneously influence the output (Sutskever et al., 2014). Convolutional neural networks (CNNs) have also been used for processing sequences because a kernel could be used to detect a feature at any position in the input sequence. However, both RNNs and CNNs exhibit poor scaling properties, leading to difficulties in handling data with long sequences (Werbos, 1990; Kolen and Kremer, 2010). To combat this limitation, transformer models (Vaswani et al., 2017), use an attention mechanism that allows the network to process an entire sequence in a fixed number of operations while maintaining temporal dependence—future information is not available in the past. Because the network is designed for a fixed number of operations, its maximum sequence length—or context window—is inherently limited. Multiple solutions have been proposed to circumvent this limit (Ren et al., 2022; Hatamizadeh et al., 2023), but these solutions all aim to implement sequential processing on a model lacking temporal complexity.

In contrast, biological neuron dynamics show a wide range of temporal complexity. In addition to the heterogeneity of neural dynamics discussed in Section 2, the spatially distributed morphology of biological neurons gives rise to temporal delays that offer an additional dimension of encoding information: temporal ordering. A single-compartment neuron model is sensitive to the timing between incoming synaptic signals but not their order. In contrast, the aforementioned tapered geometry of biological dendrites allows a distinction between the stimulation of two synapses in the forward direction compared to the reverse direction, corresponding to a distinct temporal order.

Nease et al. (2012) first demonstrated a mapping of the cable model to reconfigurable analog CMOS blocks on a computing architecture known as the Field-Programmable Analog Array (FPAA). The device uses floating gates to set a switch matrix and control the flow of currents between computational analog blocks (CAB)—see Basu et al. (2010) for more details. Using these CABs, the architecture was able to accurately replicate the dynamics of a passive length of dendrite within its linear regime and also demonstrate favorable computational properties in the nonlinear regime. George et al. (2013) applied this architecture toward modeling a Hidden Markov Model (HMM) for word spotting. In this demo, the tapered effect of the dendrite was also modeled, allowing for the detection of syllables in a word only when presented with the correct sequential ordering. Because these temporal dependencies were computed using the passive transmission properties of the dendrite cable, the devices showed >1, 000 × improvement in multiply-and-accumulate operations per Watt (MACs/W)—when compared to an equivalent HMM implemented on a digital system.

Boahen (2022) proposed a similar dendritic architecture in which several ferroelectric domains control the gate of a transistor. Under this architecture, the ferroelectric domains align only when a sequence arrives in the correct order; voltage is applied at the transistor source terminal while current is read out at the drain to form a temporal order detector, much like the dendrite. Boahen also argues that sequential encodings can sparsify communication because each pulse in a layer of *N* dendritic units represents a base-*N* digit and thus conveys *log*_2_(*N*) bits. As a result, Boahen argues that this architecture reduces the heat generated by an on-chip network and provides a more suitable architecture for 3D integrated electrical circuits.

These devices show how dendritic models provide a new dimension for encoding and decoding information that can reduce the power constraints of neural networks. In each of these examples, however, it is assumed that the dendrite is deliberately programmed to be selective to some sequence. Neither of the two architectures describes a method for learning or training the sequential encoding.

## 4 Discussion

An optoelectronic approach to neuromorphic computing is better suited to provide the interconnect bandwidths necessary to support the neuronal fan-in and fan-out required to model neural networks at biological scales while also allowing for flexible and programmable neural dynamics. The density of optoelectronic neurons may be limited due to the relatively larger scale of photonic devices compared to CMOS circuits. However, because neurons with dendritic trees are functionally similar to a two-layer neural network, the incorporation of a CMOS dendrite network would counteract these limitations by providing increased expressivity to each neuron. Additionally, a dendritic tree offers additional architectural flexibility to represent high-fan-in, low-fan-out functional units as dendritic compartments while low-fan-in, high-fan-out units are represented as neuron somas. As a result, an optoelectronic, dendritic-computing architecture is likely the key to advances in large-scale neuromorphic computing.

Figure 2 shows a diagram of an optoelectronic chiplet architecture that captures the advantages of neural heterogeneity and dendritic structures. A programmable electronic switch matrix connects analog blocks that model active and passive dendritic compartments. A photonic matrix multiplier—such as a Mach-Zehnder Interferometer mesh—forms the receiving synaptic mesh and serves as input to a number of dedicated leaf nodes containing photodetectors. Each dendritic tree would also contain a dedicated root node that models the soma and drives a laser output. Each chiplet would contain several multi-compartment optoelectronic neurons and be coupled through a shared photonic-electronic interposer, which provides a routing mesh between many chiplets. Using this architecture, neural networks could be emulated with much greater biological accuracy and at much lower power than existing neuromorphic solutions.

This optoelectronic approach to heterogeneous, dendritic neuromorphic computing would make the vision of brain-scale neuromorphic computing more feasible. However, this architecture relies on the development of packaging methods for 3D photonic and electronic integrated circuits, though an increasing number of challenges for scaling contemporary electronic systems is likely to provide a shared motivation toward the development of such integration methods. Alongside these packaging methods, more work is needed to determine an optimal number of dendritic compartments and an optimal analog model that concisely captures all of the relevant membrane dynamics of the neuron.

## 5 Conclusion

Biological neural networks benefit from heterogeneous neural dynamics and dendrite morphology that have been largely unexplored in hardware accelerators. An optoelectronic approach can implement high-bandwidth communication networks *and* programmable dynamical systems to provide a “best-of-both-worlds” solution for implementing biological complexity in neuromorphic computing architectures. More work is needed to optimize the architecture and computing model however, the stark contrast in the energy efficiency of human brains compared to modern computing systems offers substantial motivations to pursue novel computing methods.

## Data availability statement

The original contributions presented in the study are included in the article/supplementary material, further inquiries can be directed to the corresponding author.

## Author contributions

LE: Writing – original draft, Writing – review & editing. MA: Writing – review & editing. HA: Writing – review & editing. Y-JL: Writing – review & editing. MB: Writing – review & editing. SY: Writing – review & editing.

## References

[B1] Acharya J. Basu A. Legenstein R. Limbacher T. Poirazi P. Wu X.. (2022). Dendritic computing: branching deeper into machine learning. Neuroscience 489, 275–289. 10.1016/j.neuroscience.2021.10.001 34656706

[B2] Agarwal S. Jacobs-Gedrim R. B. Bennett C. Hsia A. Heukelom M. S. V. Hughart D.. (2019). “Designing and modeling analog neural network training accelerators,” in 2019 International Symposium on VLSI Technology, Systems and Application, VLSI-TSA (Hsinchu). 10.1109/VLSI-TSA.2019.8804680

[B3] Averbeck B. B. Latham P. E. Pouget A. (2006). Neural correlations, population coding and computation. Nat. Rev. Neurosci. 7, 358–366. 10.1038/nrn1888 16760916

[B4] Backus J. (1978). Can programming be liberated from the von Neumann style? Commun. ACM 21, 613–641. 10.1145/359576.359579

[B5] Balle S. Figueiredo J. M. L. Javaloyes J. Romeira B. Ironside C. N. Piro O.. (2013). Excitability and optical pulse generation in semiconductor lasers driven by resonant tunneling diode photo-detectors. Opt. Express 21, 20931–20940. 10.1364/OE.21.020931 24103966

[B6] Basu A. Brink S. Schlottmann C. Ramakrishnan S. Petre C. Koziol S.. (2010). A floating-gate-based field-programmable analog array. IEEE J. Solid-State Circuits 45, 1781–1794. 10.1109/JSSC.2010.2056832

[B7] Beausoleil R. G. (2011). Large-scale integrated photonics for high-performance interconnects. J. Emerg. Technol. Comput. Syst. 7, 326–327. 10.1109/PHO.2011.6110559

[B8] Boahen K. (2022). Dendrocentric learning for synthetic intelligence. Nature 612, 43–50. 10.1038/s41586-022-05340-6 36450907

[B9] Chang P. H. Samanta A. Yan P. Fu M. Zhang Y. On M. B.. (2023). A 3D integrated energy-efficient transceiver realized by direct bond interconnect of co-designed 12 nm finfet and silicon photonic integrated circuits. J. Lightwave Technol. 41, 6741–6755. 10.1109/JLT.2023.3291704

[B10] Chelaru M. I. Dragoi V. (2008). Efficient coding in heterogeneous neuronal populations. Proc. Nat. Acad. Sci. 105, 16344–16349. 10.1073/pnas.0807744105 18854413 PMC2571028

[B11] Davies M. Srinivasa N. Lin T. H. Chinya G. Cao Y. Choday S. H.. (2018). Loihi: a neuromorphic manycore processor with on-chip learning. IEEE Micro 38, 82–99. 10.1109/MM.2018.112130359

[B12] Dayan P. Abbott L. F. (2001). Theoretical Neuroscience: Computational and Mathematical Modeling of Neural Systems. Cambridge, MA: The MIT Press.

[B13] Elman J. L. (1991). Distributed representations, simple recurrent networks, and grammatical structure. Mach. Learn. 7, 195–225. 10.1007/BF00114844

[B14] Feldmann J. Youngblood N. Karpov M. Gehring H. Li X. Stappers M.. (2021). Parallel convolutional processing using an integrated photonic tensor core. Nature 589, 52–58. 10.1038/s41586-020-03070-1 33408373

[B15] George S. Hasler J. Koziol S. Nease S. Ramakrishnan S. (2013). Low power dendritic computation for wordspotting. J. Low Power Electron. Appl. 3, 73–98. 10.3390/jlpea3020073

[B16] Hassan A. Saha S. Carusone A. C. (2023). “Fully Integrated photonic dot-product engine in 45-nm SOI CMOS for photonic computing,” in 2023 IEEE Silicon Photonics Conference (SiPhotonics) (Washington, DC: IEEE), 1–2. 10.1109/SiPhotonics55903.2023.10141931

[B17] Hatamizadeh A. Yin H. Heinrich G. Kautz J. Molchanov P. (2023). “Global context vision transformers,” in Proceedings of the 40th International Conference on Machine Learning (Honolulu, HI: PMLR), 12633–12646.

[B18] Hornik K. Stinchcombe M. White H. (1989). Multilayer feedforward networks are universal approximators. Neural Netw. 2, 359–366. 10.1016/0893-6080(89)90020-8

[B19] Huang C. Sorger V. J. Miscuglio M. Al-Qadasi M. Mukherjee A. Lampe L.. (2022). Prospects and applications of photonic neural networks. Adv. Phys. X 7:1981155. 10.1080/23746149.2021.1981155

[B20] Indiveri G. Linares-Barranco B. Hamilton T. J. van Schaik A. Etienne-Cummings R. Delbruck T.. (2011). Neuromorphic silicon neuron circuits. Front. Neurosci. 5:9202. 10.3389/fnins.2011.00073 21747754 PMC3130465

[B21] Izhikevich E. M. (2003). Simple model of spiking neurons. IEEE Trans. Neural Netw. 14, 1569–1572. 10.1109/TNN.2003.820440 18244602

[B22] Koch C. (1984). Cable theory in neurons with active, linearized membranes. Biol. Cybern. 50, 15–33. 10.1007/BF00317936 6324889

[B23] Koch C. Laurent G. (1999). Complexity and the nervous system. Science 284, 96–8. 10.1126/science.284.5411.96 10102826

[B24] Kolen J. F. Kremer S. C. (2010). “Gradient flow in recurrent nets: the difficulty of learning longterm dependencies,” in A Field Guide to Dynamical Recurrent Networks, eds S. C. Kremer, and J. F. Kolen (New York, NY: IEEE Press), 237–374. 10.1109/9780470544037

[B25] Lee Y.-J. On M. B. Srouji L. E. Zhang L. Abdelghany M. Yoo S. B.. (2024). Demonstration of programmable brain-inspired optoelectronic neuron in photonic spiking neural network with neural heterogeneity. J. Lightwave Technol. 1–12. 10.1109/JLT.2024.3368450. [Epub ahead of print].

[B26] Liang D. Huang X. Kurczveil G. Fiorentino M. Beausoleil R. G. (2016). Integrated finely tunable microring laser on silicon. Nat. Photonics 10, 719–722. 10.1038/nphoton.2016.163

[B27] Lipton Z. C. Berkowitz J. Elkan C. (2015). A critical review of recurrent neural networks for sequence learning. arXiv [preprint]. arXiv:1506.00019. 10.48550/arXiv.1506.00019

[B28] Lu Z. Pu H. Wang F. Hu Z. Wang L. (2017). “The expressive power of neural networks: a view from the widt,” in Advances in *Neural Information Processing Systems, Vol. 30* (Long Beach, CA), 1097–1105.

[B29] Marsat G. Maler L. (2010). Neural heterogeneity and efficient population codes for communication signals. J. Neurophysiol. 104, 2543–55. 10.1152/jn.00256.2010 20631220

[B30] Mead C. (1990). Neuromorphic electronic systems. Proc. IEEE 78, 1629–1636. 10.1109/5.58356

[B31] Mercioni M. A. Holban S. (2023). “A brief review of the most recent activation functions for neural networks,” in 2023 17th International Conference on Engineering of Modern Electric Systems, EMES (Oradea). 10.1109/EMES58375.2023.10171705

[B32] Migliore M. Cannia C. Lytton W. W. Markram H. Hines M. L. (2006). Parallel network simulations with NEURON. J. Comput. Neurosci. 21, 119–129. 10.1007/s10827-006-7949-5 16732488 PMC2655137

[B33] Miller D. A. (2000). Rationale and challenges for optical interconnects to electronic chips. *Proc*. IEEE 88, 728–749. 10.1109/5.867687

[B34] Miller D. A. (2009). Device requirements for optical interconnects to silicon chips. Proc. IEEE 97, 1166–1185. 10.1109/JPROC.2009.2014298

[B35] Nease S. George S. Hasler P. Koziol S. Brink S. (2012). Modeling and implementation of voltage-mode CMOS dendrites on a reconfigurable analog platform. IEEE Trans. Biomed. Circuits Syst. 6, 76–84. 10.1109/TBCAS.2011.2163714 23852747

[B36] Orchard G. Frady E. P. Rubin D. B. D. Sanborn S. Shrestha S. B. Sommer F. T.. (2021). “Efficient neuromorphic signal processing with Loihi 2,” in IEEE Workshop on Signal Processing Systems, SiPS: Design and Implementation (Coimbra), 254–259. 10.1109/SiPS52927.2021.00053

[B37] Perez-Nieves N. Leung V. C. Dragotti P. L. Goodman D. F. (2021). Neural heterogeneity promotes robust learning. Nat. Commun. 12, 1–9. 10.1038/s41467-021-26022-3 34608134 PMC8490404

[B38] Poirazi P. Papoutsi A. (2020). Illuminating dendritic function with computational models. Nat. Rev. Neurosci. 21, 303–321. 10.1038/s41583-020-0301-7 32393820

[B39] Prucnal P. R. Shastri B. J. de Lima T. F. Nahmias M. A. Tait A. N. (2016). Recent progress in semiconductor excitable lasers for photonic spike processing. Adv. Opt. Photonics 8:228. 10.1364/AOP.8.000228

[B40] Rakowski M. Meagher C. Nummy K. Aboketaf A. Ayala J. Bian Y.. (2020). 45nm “CMOS - silicon photonics monolithic technology (45CLO) for next-generation, low power and high speed optical interconnects,” in Optical Fiber Communication Conference (OFC) 2020, (Washington, DC: Optica Publishing Group), T3H.3. 10.1364/OFC.2020.T3H.3

[B41] Ramey C. (2020). “Silicon photonics for artificial intelligence acceleration: Hotchips 32,” in 2020 IEEE Hot Chips 32 Symposium, HCS 2020 (Palo Alto, CA). 10.1109/HCS49909.2020.9220525

[B42] Ranganathan P. (2020). Technical perspective: asic clouds: specializing the datacenter. Commun. ACM 63, 102–102. 10.1145/3399738

[B43] Rasamoelina A. D. Adjailia F. Sincak P. (2020). “A review of activation function for artificial neural network,” in SAMI 2020 - *IEEE 18th World Symposium on Applied Machine Intelligence and Informatics, Proceedings* (Herlany), 281–286. 10.1109/SAMI48414.2020.9108717

[B44] Ren P. Li C. Wang G. Xiao Y. Du Q. Liang X.. (2022). “Beyond fixation: dynamic window visual transformer,” in Proceedings of the IEEE/CVF Conference on Computer Vision and Pattern Recognition (CVPR) (New Orleans, LA: IEEE), 11987–11997. 10.1109/CVPR52688.2022.01168

[B45] Shamir M. Sompolinsky H. (2006). Implications of neuronal diversity on population coding. Neural Comput. 18, 1951–1986. 10.1162/neco.2006.18.8.1951 16771659

[B46] Solli D. R. Jalali B. (2015). Analog optical computing. Nat. Photonics 9, 704–706. 10.1038/nphoton.2015.208

[B47] Spruston N. (2008). Pyramidal neurons: dendritic structure and synaptic integration. Nat. Rev. Neurosci. 9, 206–221. 10.1038/nrn2286 18270515

[B48] Stevens C. F. Wang Y. (1994). Changes in reliability of synaptic function as a mechanism for plasticity. Nature 371, 704–707. 10.1038/371704a0 7935816

[B49] Stimberg M. Brette R. Goodman D. F. (2019). Brian 2, an intuitive and efficient neural simulator. Elife 8:e47314. 10.7554/eLife.47314.028 31429824 PMC6786860

[B50] Sutskever I. Vinyals O. Le Q. V. (2014). “Sequence to sequence learning with neural networks,” in Advances in Neural Information Processing Systems, Vol. 27 (Montréal, QC).

[B51] Tait A. N. Nahmias M. A. Tian Y. Shastri B. J. Prucnal P. R. (2014). Photonic Neuromorphic Signal Processing and Computing. Berlin, Heidelberg: Springer, 183–222. 10.1007/978-3-642-40224-1_8

[B52] Tait A. N. Prucnal P. R. Shastri B. J. de Lima T. F. Nahmias M. A. (2015). Excitable laser processing network node in hybrid silicon: analysis and simulation. Opt. Express 23, 26800–26813. 10.1364/OE.23.026800 26480191

[B53] Thagard P. (2002). How molecules matter to mental computation. Philos. Sci. 69, 429–446. 10.1086/342452

[B54] Theis T. N. Wong H.-S. P. (2017). The end of Moore's law: a new beginning for information technology. Comput. Sci. Eng. 19, 41–50. 10.1109/MCSE.2017.29

[B55] Vaswani A. Brain G. Shazeer N. Parmar N. Uszkoreit J. Jones L.. (2017). “Attention is all you need,” in Advances in Neural Information Processing Systems (NIPS 2017), Vol. 30 (Long Beach, CA).

[B56] Werbos P. J. (1990). Backpropagation through time: what it does and how to do it. Proc. IEEE 78, 1550–1560. 10.1109/5.58337

[B57] Zeng H. Sanes J. R. (2017). Neuronal cell-type classification: challenges, opportunities and the path forward. Nat. Rev. Neurosci. 18, 530–546. 10.1038/nrn.2017.85 28775344

[B58] Zhang Y. Samanta A. Shang K. Yoo S. J. B. (2020). Scalable 3D silicon photonic electronic integrated circuits and their applications. IEEE J. Sel. Top. Quantum Electron. 26, 1–10. 10.1109/JSTQE.2020.2975656

